# Can You Catch a Liar? How Negative Emotions Affect Brain Responses when Lying or Telling the Truth

**DOI:** 10.1371/journal.pone.0059383

**Published:** 2013-03-25

**Authors:** Alice Mado Proverbio, Maria Elide Vanutelli, Roberta Adorni

**Affiliations:** Department of Psychology, University of Milano-Bicocca, Milan, Italy; University of Rome, Italy

## Abstract

The capacity to deceive others is a complex mental skill that requires the ability to suppress truthful information. The polygraph is widely used in countries such as the USA to detect deception. However, little is known about the effects of emotional processes (such as the fear of being found guilty despite being innocent) on the physiological responses that are used to detect lies. The aim of this study was to investigate the time course and neural correlates of untruthful behavior by analyzing electrocortical indexes in response to visually presented neutral and affective questions. Affective questions included sexual, shameful or disgusting topics. A total of 296 questions that were inherently true or false were presented to 25 subjects while ERPs were recorded from 128 scalp sites. Subjects were asked to lie on half of the questions and to answer truthfully on the remaining half. Behavioral and ERP responses indicated an increased need for executive control functions, namely working memory, inhibition and task switching processes, during deceptive responses. Deceptive responses also elicited a more negative N400 over the prefrontal areas and a smaller late positivity (LP 550–750 ms) over the prefrontal and frontal areas. However, a reduction in LP amplitude was also elicited by truthful affective responses. The failure to observe a difference in LP responses across conditions likely results from emotional interference. A swLORETA inverse solution was computed on the N400 amplitude (300–400 ms) for the dishonest – honest contrast. These results showed the activation of the superior, medial, middle and inferior frontal gyri (BA9, 11, 47) and the anterior cingulate cortex during deceptive responses. Our results conclude that the N400 amplitude is a reliable neural marker of deception.

## Introduction

When we lie (i.e., deliberately utter a falsehood with the intention to deceive), our brain arousal level is increased because of a catecholaminic response that is triggered by the Autonomic Nervous System. This system is also responsible for other body changes that can be detected easily by lie detector tests, including voice modulation, which can be detected via “*voice stress analyzers*” [1]; pupil mydriasis; increases in respiratory and cardiac frequency; and skin conductance changes (electrodermal response). However, these physiological indexes reflect an emotional perturbation rather than the cognitive act of lying. Therefore, these indexes cannot be used reliably to identify deception if an innocent suspect experiences these physiological changes due to fear.

The polygraph is widely used in countries such as the USA to detect deception. However, little is known about the effect of negative emotional processes (e.g., fear) on the physiological responses that are used to detect lies. Therefore, the aim of this study was to investigate the time course and neural correlates of untruthful behavior by analyzing electrocortical indexes in response to visually presented neutral and affective questions.

Several neuroimaging studies have revealed the crucial roles of the prefrontal and inferior frontal cortices [2] as well as the anterior cingulate cortex in the monitoring of conflict [3], the inhibition of competing responses [4], working memory [5] and the regulation of arousal [6]. These processes are all necessary to improvise false responses. For example, in an fMRI study by Ganis et al. [7], subjects were asked to lie about memorable autobiographic experiences. These lies were associated with the activation of the anterior region of the bilateral middle frontal gyrus (BA10) and the anterior cingulate cortex (BA32). In another study by Nuñez et al. [8], subjects were asked to either lie or tell the truth about either a personal experience or shared semantic information. This was associated with an increased activation of the anterior cingulate cortex, the caudate and thalamic nuclei, and the dorsolateral prefrontal cortex (DLPFC). A more recent fMRI study by Lee et al. [9] investigated the interaction between answering untruthfully and the affective valence of the subject of the lie. Based on an initial rating of the affective value of IAPS images (The International Affective Picture System), the 40 images that were judged as the most positive and the 40 images that were judged as the most negative by each participant were selected as stimuli during their experiment. Subsequently, participants were instructed to either lie or tell the truth about how they rated each picture. Lying was associated with an increase in the BOLD signal in the medial and superior frontal gyri (BA9), the left DLPFC (BA46), the cingulate cortex, the bilateral insula (BA47, 48), and the left precentral gyrus (BA9), as well as other brain regions. A clear valence-related effect on deception was observed in several brain regions, including the lateral prefrontal and inferior parietal cortex. However, activity in these regions has also been reported in fMRI studies on deception that used neutral stimuli. Therefore, the specific role of emotion in modulating brain activity during lying was not assessed conclusively in this study. A more specific effect of emotional state on deception was found in a PET study by Abe et al. [10] in which participants were asked to respond verbally with a single word to 48 questions that related to autobiographical semantic information. Deception increased the activation of the left ventromedial prefrontal cortex (VMPFC, BA11), the right medial temporal gyrus (BA38), the right inferior temporal gyrus (BA20/38) and the left amygdala, as well as other brain regions. The activation of the amygdala is compatible with its role in the perception and expression of fear sensation [11]. The role of the dorsolateral prefrontal cortex (DLPFC) was targeted in a Transcranial Direct Current Stimulation (tDCS) study by Priori et al. [12] in which subjects were asked about the possession of selected image cards. This study showed that stimulation of the DLPFC significantly disrupted the ability to lie compared with telling the truth.

These studies all required the execution of a response that was incompatible with the truth, which stimulates the activation of frontal and prefrontal cortical regions. A meta-analysis [13] of the results from 12 functional MRI and PET studies identified the activated regions that were common across all the studies during the act of lying. Working memory, which is associated with enhanced activity in the DLPFC, is important for maintaining a representation of the truth. This area is also involved in planning, problem solving, action implementation and inhibition, manipulation of information and control of emotions [8], [14], [15]. The effectiveness of a lie requires the intervention of control processes to efficiently resolve the conflict between a tendency to respond sincerely and the need to produce a mendacious response while inhibiting undesirable responses. According to neuroimaging data, the area predominantly involved in this function is the anterior cingulate cortex (ACC).

One problem with available neurometabolic studies is that these studies require subjects to lie about specific sensory material (such as pictures [16] or word lists [15]) during brain scans. Therefore, the activation data and BOLD signals represent the neural mechanisms that underlie not only the ability to lie but also the processing of the objects or sensory information that are presented (e.g., images, pictures, words). These latter brain processes are independent of deception [17]. The lack of temporal resolution in PET and fMRI techniques prevents the discrimination between the timing of the perceptual and cognitive processing of presented material and the timing of the decision making and planning and execution of untruthful vs. truthful responses.

This problem can be addressed using electrophysiological techniques such as Event-Related Potentials (ERPs). Because of their high temporal resolution, ERPs can provide data on a time scale of ms, which is the time course of the neural processing that is involved in deception processes (e.g., [15], [18]–[19].

In a recent ERP study [20], ERPs were recorded while participants responded either truthfully or untruthfully about their preferences for celebrities, food, sports or animals. The ERP results showed an increase in negativity over the fronto-central areas between 400 and 700 ms during untruthful responses. The Principal Component Analysis (PCA) for the lie - honesty activity difference identified two main dipoles in the medial frontal gyrus and middle temporal gyrus. The authors hypothesized that these areas might reflect conflict detection and control processes during the processing of false answers. In another study by Dong et al. [21], a group of students assessed the attractiveness of the individuals in 200 photographs (100 women and 100 men) using a multiple choice questionnaire. Based on the questionnaire results, 80 photographs that were rated as attractive and unattractive were selected as stimuli for the ERP recording. Subjects were asked to either lie or answer honestly about the attractiveness of the individual in each picture. The ERP data showed an increase in the LPC (Late Positive Component) between 300 and 500 ms during truthful responses compared with mendacious responses and an enhanced negativity between 500 and 1000 ms for untruthful responses. Hu et al. [22] asked subjects to respond truthfully or untruthfully about autobiographical information, such as name, date and city of birth, that could belong to either themselves or a hypothetical stranger. Deception was associated with an increased negativity (at the level of the parietal-occipital N1 and the frontal-central N2) and a decreased frontal-central P3 positivity. The effect of emotional factors on deception was not investigated in these ERP studies.

The aim of the present study was to investigate the time course and neural correlates of untruthful responses to 296 visually presented neutral and affective questions by analyzing electrocortical indexes that were recorded from 128 scalp sites in 25 volunteers. Subjects were asked to lie for half of the questions and to answer truthfully for the remaining questions.

We wished to disentangle the effect of the cognitive act of lying (controlled by the central nervous system) from the effect of the emotional and physiological activation intrinsic to lying, which is triggered by the autonomic nervous system (as well as the limbic brain, including the amygdala), by identifying reliable neural electrophysiological markers of lying and emotional states. We also wished to distinguish the brain activation related to the processing of the questions from the act of lying (or being truthful). To accomplish this latter goal, EEG was recorded in a continuous modality, and brain activity was time-locked to the response prompt that followed each question rather than to the question onset.

Based on the previous ERP literature, we expected to find an increased N400 to untruthful responses and an increased P3/late positivity to truthful responses. In addition, we investigated the effect of emotion on the two conditions (to simulate the stressful conditions under which a suspect takes a lie detector test) by asking neutral vs. embarrassing, shameful or disgusting questions. Since the effect of emotion in lying had not been previously investigated, specifically with ERPs, we had no a priori hypothesis about the possible component to be affected by it. We aimed at elucidating precisely to which extent an emotional state (very likely affecting more the healthy than the psychopathic brain) was able to mask or alter the neural marker of deception, as for example the Late Positivity described in Dong et al.’s [21] study, or the P3 from Hu et al’s [22] study.

## Materials and Methods

### Participants

Twenty-five university students (12 males and 13 females) volunteered for this experiment. The females ranged in age from 20 to 26 years (mean age = 23.15 years, SD = 1.63) and had a high level of education (15.31 years in school, SD = 2.06). The males ranged in age from 24 to 29 years (mean age = 24.83 years, SD = 1.85) with the same level of education as the females (15.25 years in school, SD = 2.14). All participants had normal or corrected-to-normal vision with right eye dominance. All participants were right-handed as assessed by the Edinburgh Inventory, and none had any left-handed relatives. Experiments were conducted with the understanding and written consent of each participant according to the Declaration of Helsinki (BMJ 1991; 302: 1194) with approval from the Ethical Committee of the Italian National Research Council (CNR) and in compliance with APA ethical standards for the treatment of human volunteers (1992, American Psychological Association). All participants received academic credit for their participation. Data from 2 men and 2 women were subsequently discarded because of excessive eye movements and electroencephalogram (EEG) artifacts.

### Stimuli

The stimuli were selected from an initial set of 320 sentences that were evaluated by a group of 20 judges (10 men and 10 women) for their affective content on a 3-point scale (not at all, somewhat emotional, extremely emotional). The sentences were posed in the form of questions relative to inherently true or false facts. For example, a few questions that were used in the study include the following: <<Is Washington, D.C. the capital of the United States?>> (True sentence) Neutral. <<Does the eel live in the desert?>> (False sentence) Neutral. <<Have you ever tortured a child to death?>> (False sentence) Emotional. <<Have you ever put your fingers in your nose? (True sentence)>> Emotional. Neutral sentences evaluated by more than 40% of judges as emotional were discarded from the initial set of stimuli. Likewise, emotional sentences evaluated by more than 40% of judges as neutral were discarded. The final set of stimuli included 296 neutral and affective questions that were typed in Arial narrow size 10 font and balanced for topic, type of information (semantic or autobiographical knowledge), affective value (within the true and false categories), and length (min = 26.6 letters, 5.16 words; max = 32.3 letters, 5.57 words; mean = 29.2 letters, 5.35 words) across all categories (74 true neutral questions; 74 true affective questions; 74 false neutral questions; 74 false affective questions). Sentences were presented at a visual angle of approximately 3° 24′ (min = 1° 30′, max = 6°) in length and 1° 28′ (min = 30′; max = 1° 45′) in height.

Each sentence was presented for 1400 ms in one or two short lines around the fixation point. Following an inter-stimulus interval (ISI) that ranged from 500 to 600 ms, a red cross (1 cm in size, 0.5 degree of visual angle) appeared at the center of the visual field for 2 seconds to prompt the motor response. The EEG was synchronized to the onset of the response prompt.

### Task and Procedure

Participants were seated comfortably in a dark and acoustically and electrically shielded test area in front of a high-resolution computer screen located 114 cm from their eyes. Participants were instructed to gaze at the center of the screen at a small red circle that served as the fixation point and to avoid any eye or body movements during the recording session.

The task consisted of responding to questions as quickly and accurately as possible by pressing a response key with the index or middle finger (yes or no, respectively) according to the specific instructions (lie vs. answer truthfully). The two hands were alternated during the recording session. The order of the hand and task conditions was counterbalanced across subjects. At the beginning of each session, subjects were told what the task requirement was (lying or telling the truth) and which hand would be used to make responses. For each condition, 2 stimuli sequences (or runs) were presented one for each response hand, separated by a short pause. Overall the experimental session comprised the presentation of 8 runs. The experimental session was preceded by a training session that included two conditions: lie or answer truthfully, for each of the two hands (i.e. 4 short stimuli sequences).

### EEG Recording and Analysis

EEG data were recorded continuously from 128 scalp sites at a sampling rate of 512 Hz using the EEProbe recording system (Advanced Neuro Technology (ANT) Enschede, The Netherlands).

Horizontal and vertical eye movements were also recorded using the linked ears as the reference lead. The EEG and electrooculogram (EOG) were amplified with a half-amplitude band pass of 0.016–100 Hz. Electrode impedance was maintained below 5 kΩ. EEG epochs were synchronized with the onset of the stimulus presentation. Computerized artifact rejection was performed to discard epochs in which eye movements, blinks, excessive muscle potentials or amplifier blocking occurred. The artifact rejection criterion was a peak-to-peak amplitude that exceeded 50 µV, which resulted in a rejection rate of ∼5%. Evoked-response potentials (ERPs) from 100 ms before (−100 ms) to 1000 ms after stimulus onset were averaged. ERP components (including the site and latency to reach maximum amplitude) were identified and measured with respect to the average baseline voltage over the interval from −100 to 0 ms.

The amplitudes of the N400 component, which reached its maximum amplitude between 300 and 400 ms, and the prefrontal late positivity (LP), which reached its maximum amplitude between 550 and 750 ms, were measured at anterior frontal (AF3, AF4, AFp3h, AFp4h) and prefrontal and frontocentral sites (AFF5h h, AFF6h, FFC3h, FFC4h), respectively.

Topographical voltage maps of the ERPs were generated by plotting color-coded isopotentials that were obtained by interpolating voltage values between scalp electrodes at specific latencies. A multifactorial repeated-measures analysis of variance (ANOVA) was applied to the ERP data. The factors included condition (deception, truthfulness), emotional content (emotional, neutral), question intrinsic veracity (true, false), electrode (according to the ERP component of interest) and hemisphere (left, right). Multiple post-hoc mean comparisons were performed using the Tukey test. Reaction times (RTs) that exceeded the mean value ±2 standard deviations were discarded, which resulted in a rejection rate of 5%. Error rate percentages were converted to arcsin values. Both RTs and error percentages were subjected to separate multifactorial repeated-measures ANOVAs with 3 within-subject factors: condition (deception, truthfulness), emotional content (emotional, neutral), and question intrinsic veracity (true, false).

## Results

### Behavioral Results

#### Accuracy data

**Figure 1:**
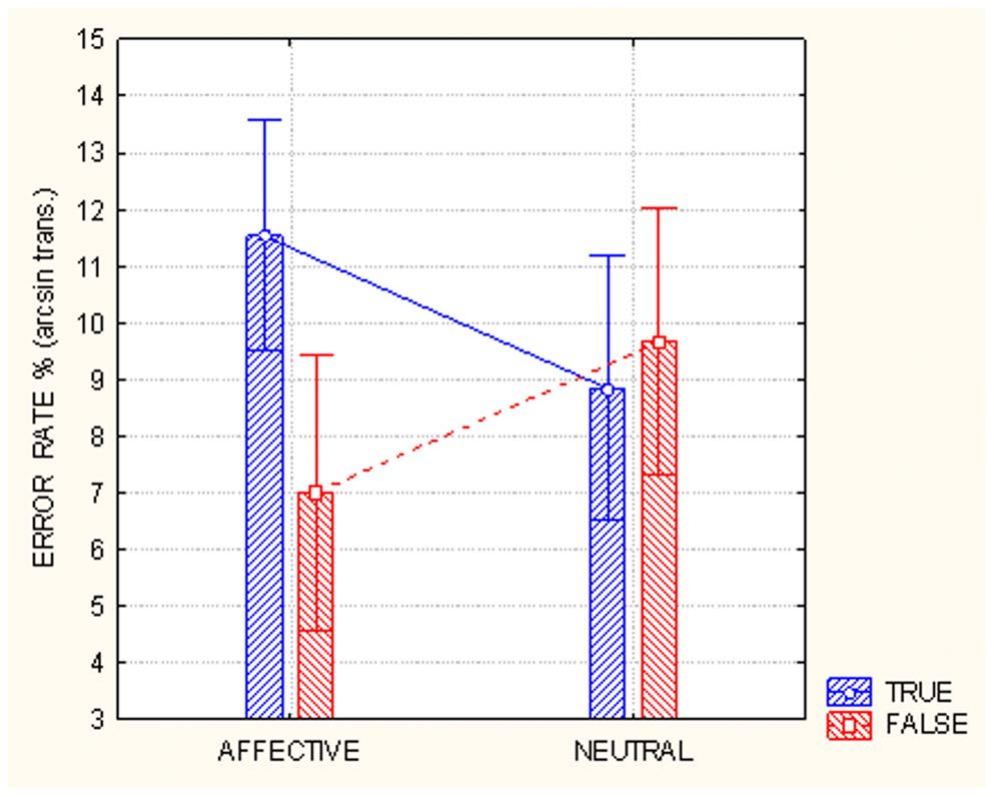
Arc sin transformed percentage of errors committed in responding to affective or neutral true or false questions. The data show how challenging was to tell the truth about an emotional question related to sex, disgusting matter or shameful behavior, while it was much easier to deny a false statement.

The analysis revealed a main effect of condition (F1, 24 = 104.07, p<0.00001) in which subjects committed more errors when they had to lie compared with when they had to answer truthfully (deception: 12.37%, SE = 0.63 and truthfulness: 6.16%, SE = 0.44). The analysis also revealed a significant effect of the question veracity (F1, 24 = 5.07, p<0.05) and post-hoc comparisons showed that the subjects committed more errors when the question was inherently true (10.18%, SE = 0.57) compared with when it was inherently false (8.34%, SE = 0.65), as displayed in Fig. 1. This effect also depended on the significant interaction between emotional content and question veracity (F1, 24 = 3.18, p<0.0005). Post-hoc comparisons showed that subjects committed more errors (p<0.05) when responding to emotional true sentences (11.53%, SE = 0.7) compared with neutral true sentences (8.84%, SE = 0.8); however, subjects committed more errors (p<0.05) on neutral false sentences (9.67%, SE = 0.8) compared with emotional false sentences (7.02%, SE = 0.83). Moreover, subjects committed more errors on emotional questions (p<0.0005) when they were true (11.52%, SE = 0.7) compared with when they were false (7.02%, SE = 0.83).

#### Response times (RTs)

**Figure 2:**
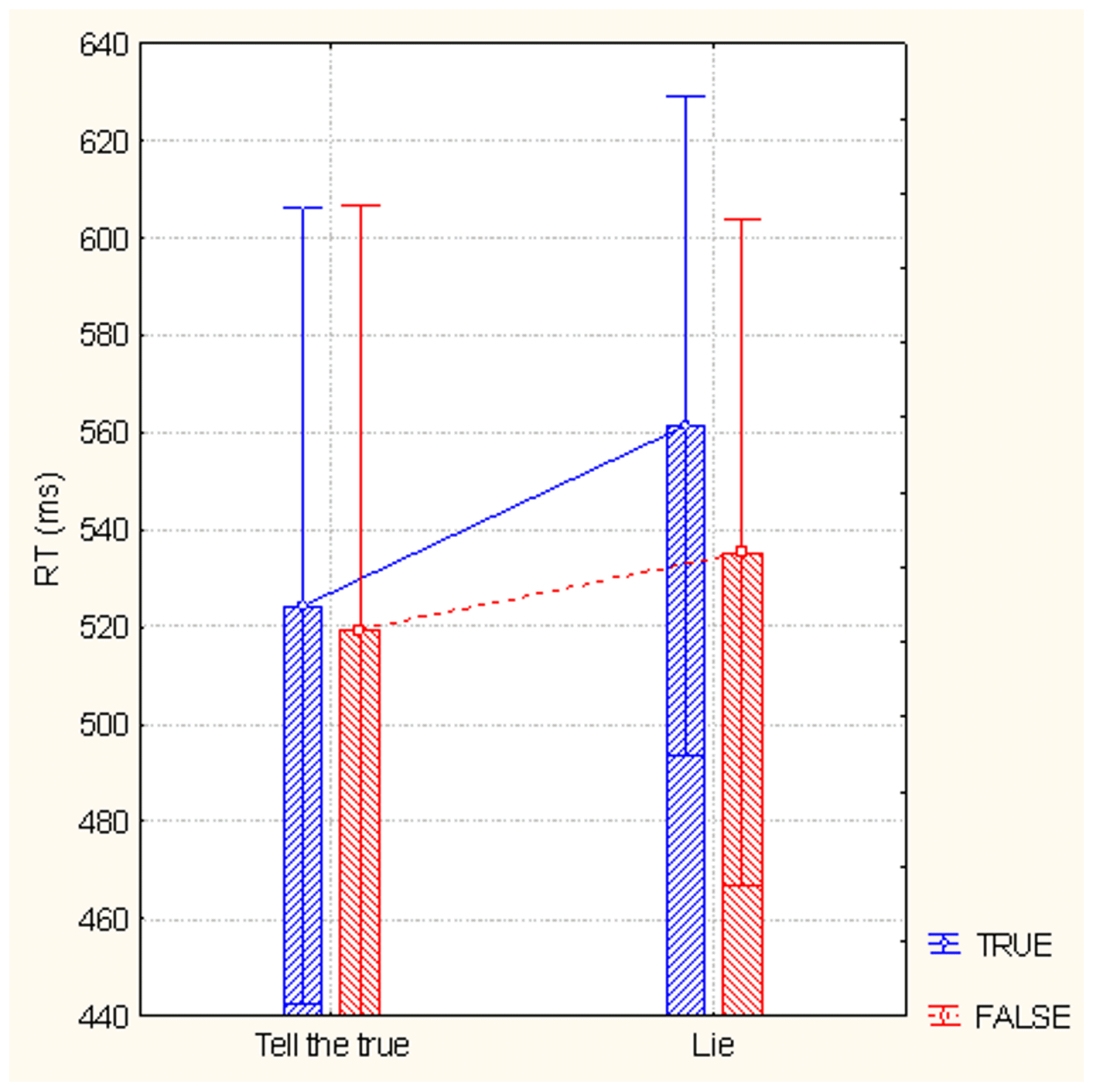
Response times recorded in response to inherently true or false statements, as a function of experimental condition (lie, or tell the truth). The data show how it was much difficult to deny a truthful than false information.

The ANOVA revealed a significant effect of condition (F1, 24 = 5.87, p<0.05) with faster responses in the truthfulness compared with the deception condition (tell the truth = 522 ms, SE = 28.79; lie = 548 ms, SE = 23.23). The emotional content of the questions also affected the RTs (F1, 24 = 6.8, p<0.05) with faster responses to neutral (527 ms, SE = 26) compared with emotional (543.35 ms, SE = 25.54) questions. Furthermore, significant effects of question veracity (F1, 24 = 16.98, p<0.0005) and the interaction of veracity and condition (F1, 24 = 4.83, p<0.05) were found. Post-hoc comparisons showed that responses were significantly slower (p<0.0005) when subjects were lying about a true (561.4 ms, SE = 23.29) compared with a false (524.34 ms, SE = 28.11) question. No significant difference was found between RTs to false questions in the two conditions (as displayed in Figure 2).

### Electrophysiological Results

**Figure 3:**
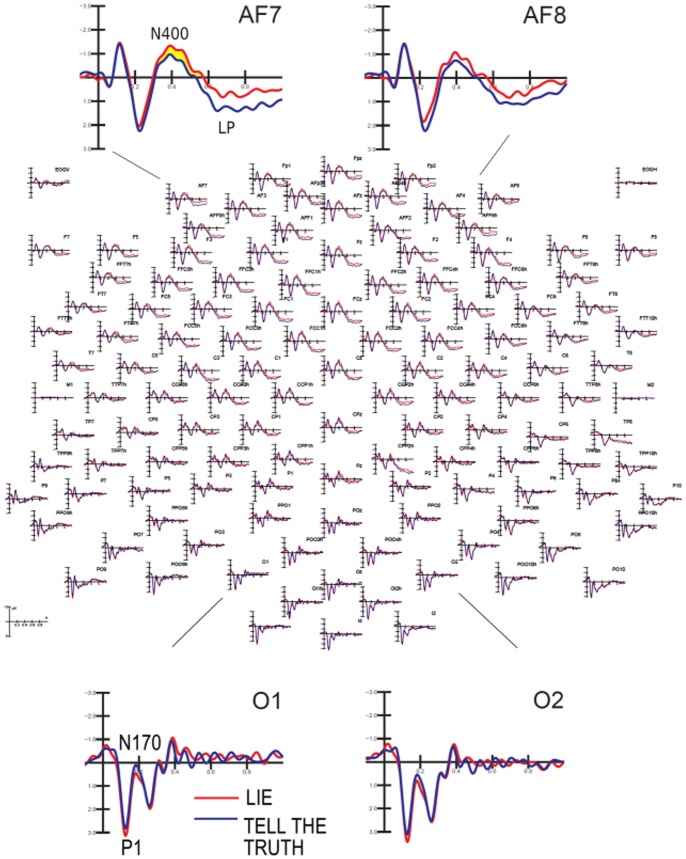
Grand-average ERPs recorded at 128 scalp sites over the left and right hemisphere during the “lie” and “tell the truth” conditions.

The ERPs recorded at anterior and posterior scalp sites in the two conditions (lie, tell the truth) are shown in Fig. 3. The two conditions differ in the amplitude of both the N400 and LP responses over the prefrontal sites. There was no difference between the two conditions observed at posterior sites, which suggests that the linguistic, perceptual and sensory nature of the questions that were posed to the subjects were identical in both conditions.

#### N400 response

The ANOVA performed on the N400 amplitudes revealed a significant effect of hemisphere (F1,20 = 5.25, p<0.05) with a stronger activation of the left compared with the right hemisphere (LH: −1 µV, SE = 0.3, RH = −0.84 µV, SE = 0.3). The largest activity was observed over the left prefrontal site, as shown by the interaction of the factors electrode and hemisphere (F1,20 = 12.56, p<0.005).

**Figure 4:**
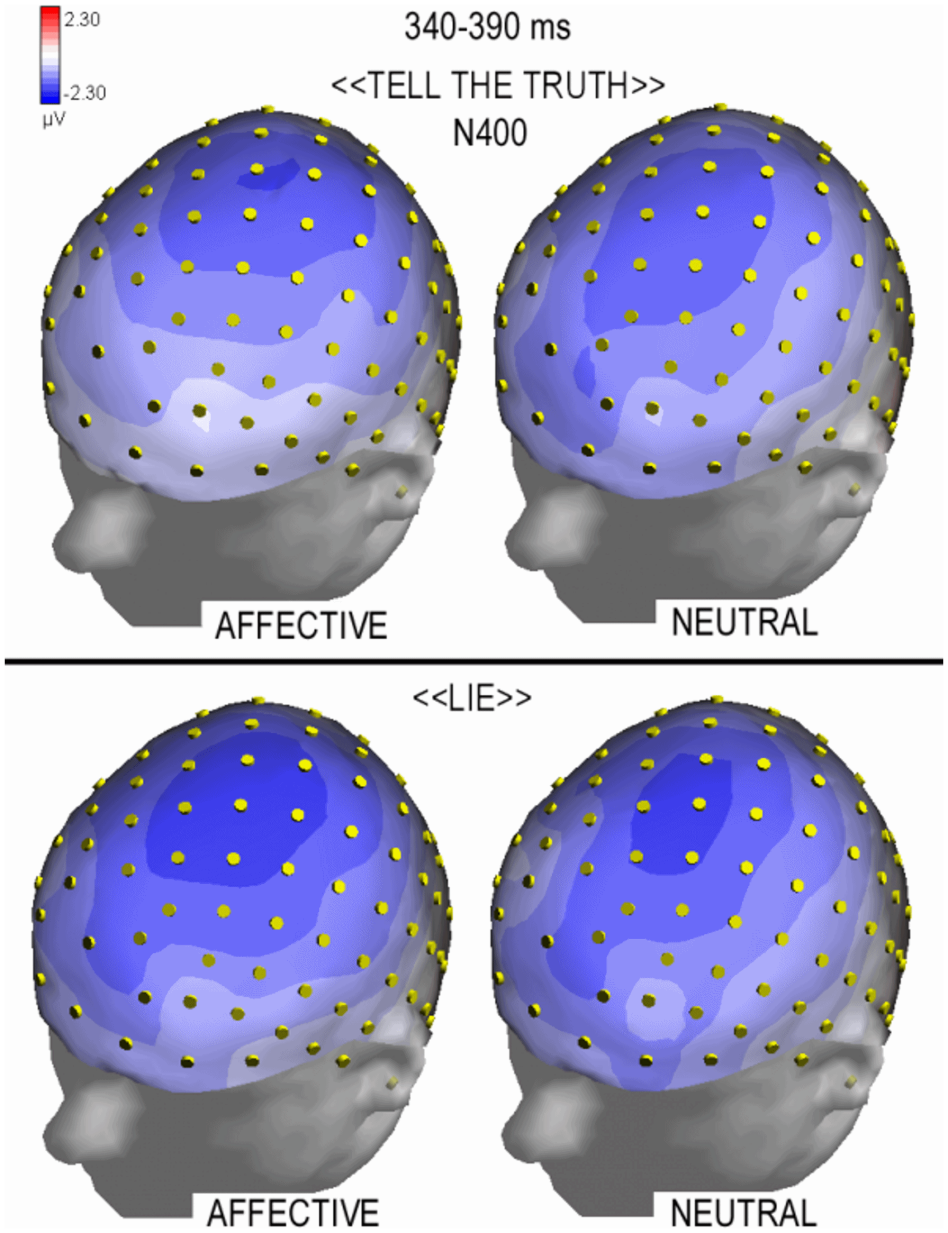
Isocolour topographic maps (top left view) of brain voltage recorded in between 340–390 ms of latency (N400 peak) during the lie (Bottom) vs. tell the truth condition (Top) as a function of question affective value (Left = affective; Right = neutral). Mendacious responses were characterized by an increase in negativity at this stage of processing.

The ANOVA also revealed a significant effect of condition (lie, tell the truth) (F1,20 = 6.95; p<0.05) in which lying was associated with a larger N400 amplitude compared with the truthful condition (lie: −1.27 µV, SE = 0.33; tell the truth: −0.57 µV, SE = 0.33). Figure 4 shows the scalp distributions of the N400 component, which was larger at central locations, compared with task-related modulation, which was larger at prefrontal sites (see Fig. 3).

**Figure 5:**
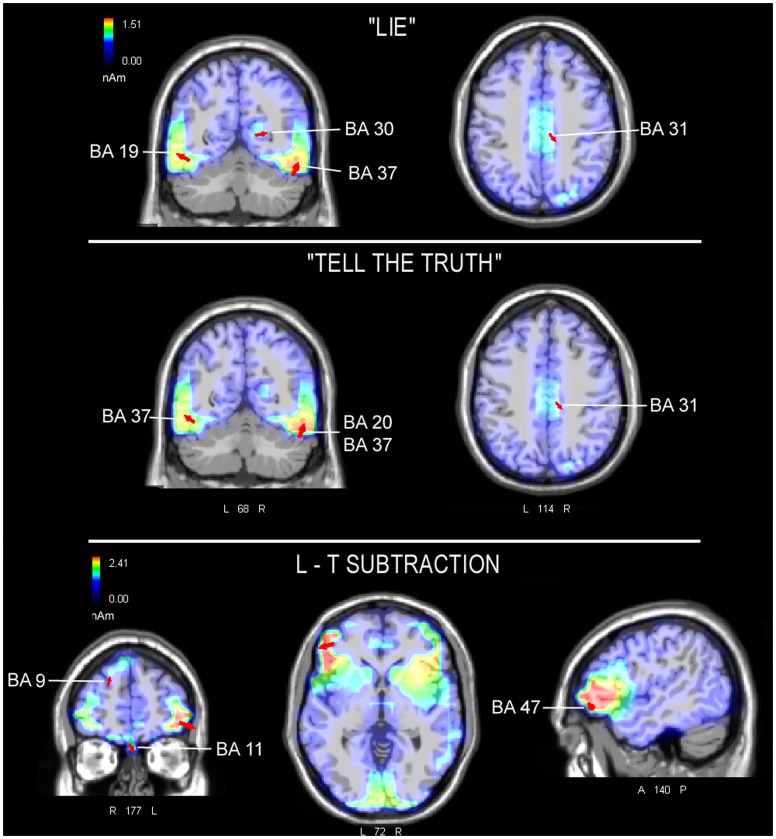
swLORETA inverse solution performed on ERP responses recorded in the two conditions and on the difference waves (“lie” minus “tell the truth” condition) in the time window of 300–400 corresponding to the N400 component. Red arrows indicate electromagnetic dipoles. Coronal, axial and sagittal views are represented (from the left). L = left hemisphere, R = right hemisphere, A = anterior, P = posterior.

To identify the neural bases of this effect, a swLORETA inverse solution (Fig. 5, Top) was applied to the ERP responses that were recorded in the lying condition between 300–400 ms post-stimulus. Table 1 reports the electromagnetic dipoles that generated the surface voltage of the N400 component. The inverse solution showed that the strongest generators of the N400 component were located bilaterally within the fusiform gyrus (BA37/19) and the right cingulate cortex (BA30 and 31). A swLORETA inverse solution was also applied to the ERPs recorded in the tell the truth condition (Fig. 5, Middle) and showed that the strongest neural generators were also located over the left and right fusiform gyrus (BA20/37) but involved less recruitment from the cingulate cortex (see the dipole magnitude in Table 1). To better highlight the differences between the lie and tell the truth conditions, difference waves (for each EEG channel) were computed between the two conditions (lie – telling the truth). A swLORETA inverse solution was applied to the difference waves in the 300–400 ms time window. The results (Fig. 5, bottom) showed that untruthful responses were associated with stronger activity in the left and right anterior brain regions (including BA47, 9, 11, and the cingulate cortex), especially the left middle frontal gyrus (BA47, see Table 2 for a list of dipoles).

**Table 1:** Tailarach coordinates (in mm) corresponding to the intracranial generators explaining the surface voltage recorded during response time in the lie vs. tell the truth condition in the 300–400 ms time window, according to swLORETA (ASA) [23], grid spacing = 5 mm, estimated SNR = 3.

Magn.	T-x (mm)	T-y (mm)	T-z (mm)	Hem.	Lobe	Area	BA
**“LIE”**							
15.09	51	−55	−18	RH	Temp	FG	37
13.49	−49	−66	−11	LH	Temp	FG	19
11.29	21	−68	5	RH	Limbic	Post. Cingulate	30
10.36	11	−30	35	RH	limbic	Cingulate gyrus	31
**“TELL THE TRUTH”**							
15.09	51	−55	−18	RH	Temp	FG	37
14.29	51	−34	−24	RH	Temp	FG	20
13.18	−49	−56	−10	LH	Temp	FG	37
11.05	21	−90	21	RH	Occ	Cuneus	18
10.38	51	−1	−28	RH	Temp	MTG	21
9.33	11	−30	35	RH	Limbic	Cingulate gyrus	31

Power RMS: Lie = 59.4; Tell the truth = 53.1 µV.

**Table 2:** Tailarach coordinates (in mm) corresponding to the intracranial generators explaining the different voltage recorded in the lie minus tell the truth conditions in the 300–400 ms time window, according to swLORETA (ASA) [23], grid spacing = 5 mm, estimated SNR = 3.

Magn.	T-x (mm)	T-y (mm)	T-z (mm)	Hem.	Lobe	Area	BA
**“LIE” - “TELL THE TRUTH”**							
31.58	−49	36	−3	LH	Frontal	Middle Frontal g.	47
24.84	41	27	−11	RH	Frontal	Inferior Frontal g.	47
21.92	2	38	−18	RH	Frontal	Medial Frontal g.	11
19.51	21	52	34	RH	Frontal	SuperiorFrontal g.	9
16.79	−19	−1	65	LH	Frontal	SuperiorFrontal g.	6
17.55	61	−6	37	RH	Frontal	Precentral gyrus	6
15.11	−9	−1	−28	LH	Limbic	Uncus	28
14.61	2	2	29	RH	Limbic	AnteriorCingulate	24
20.16	61	−55	−18	RH	Occipital	Fusiform gyrus	37
20.12	61	−25	−16	RH	Temporal	Inferior Temporal g.	20
19.98	−19	−97	−13	LH	Occipital	Lingual gyrus	18
14.86	−59	−9	−22	LH	Temporal	Inferior Temporal g.	20
22.27	11	−99	2	RH	Occipital	Cuneus	
27.11	11	−73	49	RH	Parietal	Precuneus	7

Power RMS: 10.3 µV.

Analysis also showed a significant effect of question veracity (F1,20 = 5.05, p<0.05) in which the N400 amplitude was larger during false (−1.17 µV, SE = 0.29) compared with true (−0.67 µV, SE = 0.35) questions.

Furthermore, the ANOVA showed a significant interaction between affective value and hemisphere (F1,20 = 6.49, p<0.05). Post-hoc mean comparisons showed stronger activity over the left (−1.06 µV, SE = 0.33) compared with the right (−0.8 µV, SE = 0.33) hemisphere for responses to neutral questions and no hemispheric asymmetry for responses to affective questions.

#### Late positivity (LP)

**Figure 6:**
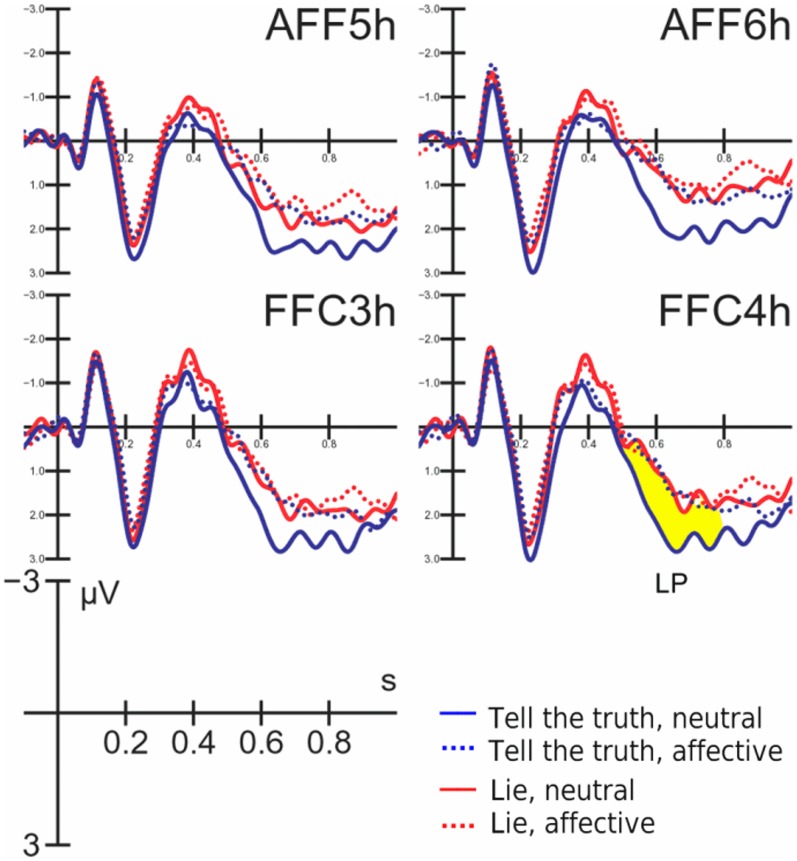
Grand-average ERP waveforms recorded at left and right anterior frontal and fronto-central sites as a function of questions affective content (dotted: affective, solid: neutral) and task conditions (blue = tell the truth; red = lie). It is visible a lack of difference in LP responses to affective questions across the two ask conditions (lying vs. telling the truth) probably because of the emotional interference. On the other hand, LP clearly differentiates the response on the basis of its truthfulness when no emotion is involved.

**Figure 7:**
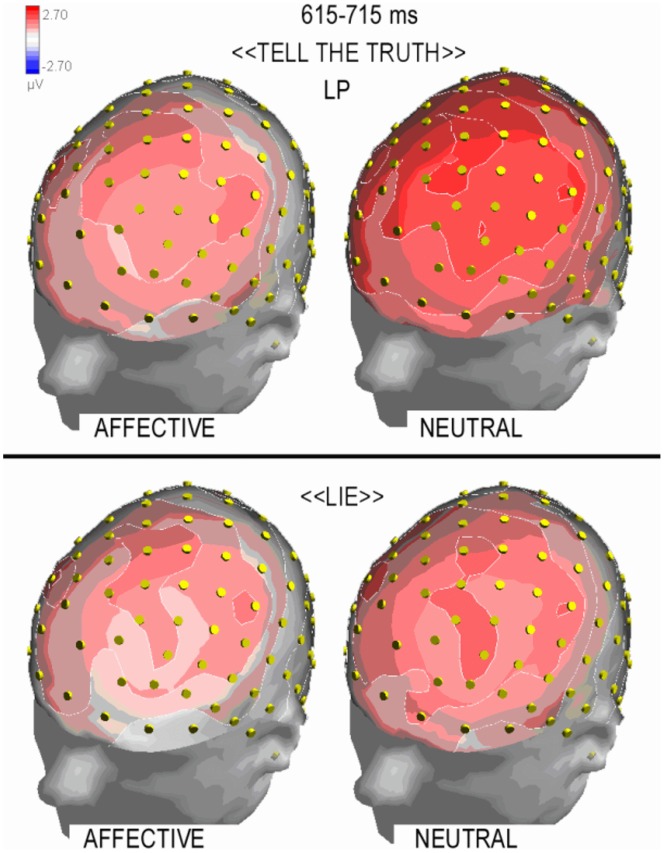
Topographical maps of surface volte activity recorded at the time window corresponding to LP maximum amplitude, as a function of questions affective content (left: affective, right: neutral) and task conditions (top = tell the truth; bottom: lie).

LP deflection reached its maximum amplitude between 550 and 750 ms over central sites (as displayed in Fig. 6). LP amplitude was quantified for task-related modulation that was observed at anterior frontal and frontal sites (AFF5h, AFF6h, FFC3h, FFC4h). Fig. 7 shows the combined effect of the condition and the affective value of the question at these locations. The ANOVA revealed a significant effect of the condition (F1,20 = 7.43; p<0.05) in which truthful responses elicited a larger LP compared with untruthful responses (1.92 µV, SE = 0.33 and 1.36 µV, SE = 0.23, respectively). Furthermore, emotional content was also significant (F1,20 = 8.53, p<0.01) such that larger LP were observed in response to neutral compared with emotional questions (1.96 µV, SE = 0.32 and 1.33 µV, SE = 0.32, respectively). The post-hoc comparison of the significant three-way interaction of condition, emotional content and hemisphere (F1,20 = 4.47, p<0.05) revealed that LP responses were larger during truthful compared with untruthful responses only when the affective content was neutral (especially over the right hemisphere). However, there was no difference in the response to emotional questions between the two conditions in either hemisphere, as displayed in Fig. 6 (LP mean amplitudes: Truthful neutral: RH = 2.29 µV, SE = 0.3; LH = 2.45 µV, SE = 0.37. Truthful emotional: RH = 1.26 µV, SE = 0.4; LH = 1.7 µV, SE = 0.42. Untruthful neutral: RH = 1.28 µV, SE = 0.25; LH = 1.8 µV, SE = 0.3. Untruthful emotional: RH = 1 µV, SE = 1.27; LH = 1.37 µV, SE = 0.36).

The similarity of the LP responses to affective questions in both conditions (lying vs. telling the truth) can be observed in the scalp topographical distributions of LP voltage depicted in Fig. 6. The failure to observe a difference in LP responses across conditions likely results from emotional interference.

## Discussion

The analysis of behavioral responses showed an increase in the time needed to respond to untruthful compared with truthful responses [8], [9], [20]–[22]. This increase in response time is also known as the “lie” effect [9]. The analysis also showed a decrease in accuracy due to both response conflict and the need to suppress truthful information. Lying requires a greater mental load due to the need to inhibit the honest response, which is more automatic, and prevaricating. Consequently, the need for “extra” cognitive resources during lying triggers the activation of control-related brain regions such as the frontal and prefrontal cortical areas [14].

The analysis of our behavioral data also showed that responses to inherently false questions were faster and more accurate than responses to inherently true questions. Subjects may find it easier to answer questions that can be immediately recognized as incorrect, such as “Is the cob a type of fungus?” compared with questions in which the truth needs to be verified, such as “Can there be raisins in a cake?”.

In addition, our analysis showed that responses to emotional questions resulted in longer RTs compared with responses to neutral questions. This finding indicates that there is a cost associated with processing affective compared with neutral information. This finding has been supported by previous neuroimaging studies [11], [24], [25] that have shown that emotionally valenced stimuli are prioritized during processing and are able to interrupt or disrupt ongoing cognitive processes and divert attention from the primary cognitive task. This property has a clear adaptive value that enables a quick reaction to potentially threatening stimuli [26]. For example, in a recent study [27] of the Stroop task with facial expressions, subjects were asked to categorize the emotions conveyed on the faces in the photographs that were shown and to ignore the words that were presented in the center of the face. These words could be neutral or affective and the results demonstrated that only the affective distracters interfered with task performance.

In the present ERP data, lying was associated with an increase in negativity (N400) between 300 and 400 ms over the prefrontal areas, especially in the left hemisphere. This finding likely indexes an increased mental workload. This increase in the N400 component, which we consider a reliable neural marker of a lie, was independent of the affective value of the question.

To identify the origin of this effect, 3 different swLORETA inverse solutions were applied to the ERP responses recorded between 300 and 400 ms during lying, telling the truth, and the difference between the two conditions to differentiate the patterns of cerebral activation. The inverse solutions showed a common activation of the ventral stream, namely the fusiform gyrus of the left (BA19 and BA37) and right (BA20 and 37) hemisphere (involved in the processing of visual objects of various categories [28], in response to the visual processing of both the sentences and the fixation red cross in both the lying and telling the truth conditions. Therefore, this result indicates that both conditions triggered the same perceptual processes.

In addition, compared with the tell the truth condition, the lie condition showed stronger activation of the posterior cingulate cortex (BA30), which is an area that is involved in the encoding of emotional aspects of visual information [29]–[32]. The swLORETA applied to the differential activity recorded in the lie minus the tell the truth condition showed significant activations in a series of anterior regions, with the strongest activation observed in the left middle frontal gyrus (BA 47); the right middle, inferior and superior frontal gyri; and the anterior cingulate cortex (BA24). Our pattern of results is consistent with the findings by Abe et al. [10], in which falsifying truthful responses was associated with increased brain activity in the left dorsolateral and right anterior prefrontal cortices. Therefore, these findings support the interpretation of previous studies that the generation of untruthful responses is related to executive function.

Prior neuroimaging studies have shown a role of the BA47 in motor response inhibition [7], emotion regulation [33] and cognitive and self-control [34]–[35]. Furthermore, the medial orbitofrontal gyrus (BA11) has been associated with the implementation of processes that underlie control performance [36]–[38], automonitoring in action regulation [39], [40], and conflict detection and control [16], [19]. This area is also involved in emotion regulation [33], [41], [42]. For example, a study by Ohira et al. [43] on the voluntary suppression of emotions showed that this area is associated with the top-down control of peripheral physiological responses that are linked to an emotional experience.

The superior frontal gyrus (BA6 and BA9), which is part of the dorsolateral prefrontal cortex (DLPFC), has been implicated in the formulation of mendacious responses [7]–[10], [12], [13] and is responsible for regulating and inhibiting undesired behavior. Studies suggest that the DLPFC plays a key role in maintaining relevant information in working memory [44], [45], inhibiting irrelevant information and responses, and trouble shooting, conflict monitoring and conflict solving [46], [47].

The activation of the premotor cortex (BA6), which was also found in studies by Ganis et al. [7] and Nuñez et al. [8], has been related to the need to suppress undesired behavior and prepare the correct motor response.

Finally, the anterior cingulate cortex (BA24) plays a multifunctional role in controlling and monitoring responses in the event of a conflict between the required answer and a more automatic but undesired answer [15], [47], [48]. This area is also involved in the inhibition of such undesirable responses [4], [36], [49], [50].

The N400 amplitude was larger over left compared with right prefrontal sites, which may be related to the linguistic nature of the stimuli that were used in this study. Some studies have shown greater activity over the right hemisphere in tasks that involve lying [51], but many of these studies have used pictures or photographs rather than phrases for the experimental stimuli. In our study, N400 was not affected by the affective valence of the stimulus. Therefore, N400 is a reliable neural marker of lying that is independent of the emotional circumstance. In contrast, the late positivity between 550 and 750 ms post-stimulus at prefrontal and frontal sites was identified as a neural marker for truthful responses. This finding is consistent with the results of many other ERP studies on the LP component (e.g., [21], [22]) and on P300 responses [52], [53]. Johnsons et al. [18] has suggested that the decrease in the amplitude of the LP for deceptive responses may be due to the inhibition of truthful answers.

However, in our study we observed that the LP did not distinguish between truthful and untruthful responses when the question was emotional. Therefore, the use of LP as a neural marker may not be reliable if the data are used as legal proof to incriminate a suspect. This finding indicates that the emotional tone of the question can modulate brain activity in relation to the responses given in the two different conditions. This finding agrees with Ekman & Sullivan [54] in which the authors stated that changes from the autonomic system are not in themselves direct measures of the lie but rather are the product of emotions. These automatic changes in the autonomic response are related to feelings of guilt and shame (as well as the fear of being discovered) and should not be considered as measures of the lie itself. The activation of the autonomic nervous system and the affective brain (in our study BA24 and BA28, limbic cortex) may affect both the LP amplitude and the physiological parameters of an extremely anxious person who is being questioned for a disgraceful crime. Therefore, the LP amplitude is not a reliable marker for deception. However, the N400 is a reliable marker of lying that is not affected by emotional factors.

### Conclusions

The ERP data show the existence of a reliable neural marker of lying in the form of an increased amplitude of the N400 component (which likely indexes conscious control processing) in frontal and prefrontal regions of the left hemisphere between 300 and 400 ms post-stimulus. Importantly, this marker was observed to be independent of the affective value of the question. The neural generators underlying this effect included the prefrontal cortex and anterior cingulate cortex. In contrast, a later LP deflection proved to be a marker of truthfulness only for neutral questions because emotional questions always reduced LP amplitudes (which likely indexes an increased arousal level that is triggered by the emotion-related autonomic response) regardless of whether the responses were truthful or untruthful.

One possible limitation of this study is that lying or telling the truth (although performed rather automatically and very accurately by anxious participants) was not specifically reinforced or guided by low-level emotional drives such as fear or pleasure (as can occur in real life), but were cognitively guided (“I must do as required”). However, the same problem holds for all ERP studies in the literature (e.g., [10], [21], [22]) as well as neuroimaging studies.

Overall, we believe that the 2 neural markers that we have discovered are sufficiently general to apply to a wider neural mechanism of lying and involve a look-for-reward or pain-avoidant motivation.
